# The Causes of Industrial Accidents

**Published:** 1919-05-31

**Authors:** 


### THE CAUSES OF INDUSTRIAL ACCIDENTS.

THERE are many and surprising factors in the causation of industrial accidents. Investigations primarily undertaken in connection with the health and efficiency of our munition workers in war-time have brought a number of interesting points to light, and the publications of the Committee responsible for these matters and proceedings of several scientific societies provide us with invaluable material upon which to found any future schemes of improved labour conditions. It is stated that annually over one thousand workers are killed by accidents in England alone, while more than 100,000 receive injuries placing them on the sick-list for a week or more. As many as two million suffer minor damage!

The time and fatigue factors are very noticeable. Accidents are at a minimum on a twelve-hour day during the first hour, and steadily increase in frequency throughout the morning. If, however, a ten-hour day is instituted, this increase, with men at least, is very much less marked, but still prominent in records of female labour. An eight-hour day or less is necessary to bring their accident-rate to the same figure as observed with the men, and, taking a generalisation, accidents are nearly three and a-half times more frequent on the twelve-hour than on the ten-hour day. Another cause is found in the speed of production. Under the system of attaining some guaranteed figure in shift output or of pieceworking, a definite hastening at the end of each spell is constantly accompanied by a corresponding crop of mishaps. Fatigue is certainly greatest at this same period, but there seems no doubt that neither factor is alone responsible.

In much of our past social work there has been a tendency to lay perhaps too much stress upon the importance of external and circumstantial conditions, and to neglect the influence from individual habits of the operatives. During the middle and later period of the war, restrictions upon the sale of alcohol had a marked effect. There was a steady diminution in that excess of accidents noticed to coincide with the first spell of night work which followed the period at which alcoholic drink was most likely to be taken. Monday was also found to possess a constantly high rate, perhaps for obvious reasons, and if a survey of any community of workers was taken it was noticed that wherever it was the habit to perform household duties, shop, or spend time in social duties before starting a particular shift in the day, so the casualty rate of that shift persistently remained the highest.

Working environment has, however, a more marked effect, but, fortunately, it is much easier to control by ordinary public-health measures, for the influence is far less insidious. Any fatigue-producing factor, whether general or affecting one bodily system in particular is of vital importance. We know that accidents in factories are at a minimum when the temperature is kept between 60° to 70° F. We now know many more physical factors. Defective lighting or local illumination produce a marked increase, but more to the face or eyes than to the hands, although damage to both occur with greater frequency.

So might one run on through a hundred causes, some outstanding and obvious, some apparently indirect and trivial, yet, when a year's figures are consulted, all are in crying need of remedy. The task before social workers has never been light, and it has rarely been clearly defined, but the wealth of observations which these past years have produced, based for the most part on unquestionable evidence, has brought a flood of light into the darkness. Not only the Government, but also those men who, directly or indirectly, can by their personal influence effect the preliminary stages in the many reforms we need, must act strongly and immediately to break the conservation of the past and open the way to the many lesser social workers who await but their opportunity.

